# Supplementary material for: Cytomegalovirus-induced inactivation of TSC2 disrupts the coupling of fatty acid biosynthesis to glucose availability resulting in a vulnerability to glucose starvation
Source: mBio. 2023 Dec 20;15(1):e03031-23. doi: 10.1128/mbio.03031-23 (PMC10790783; doi:10.1128/mbio.03031-23)
Supplement: Supplemental figures — Fig. S1 to S8. [file mbio.03031-23-s0009.pdf]

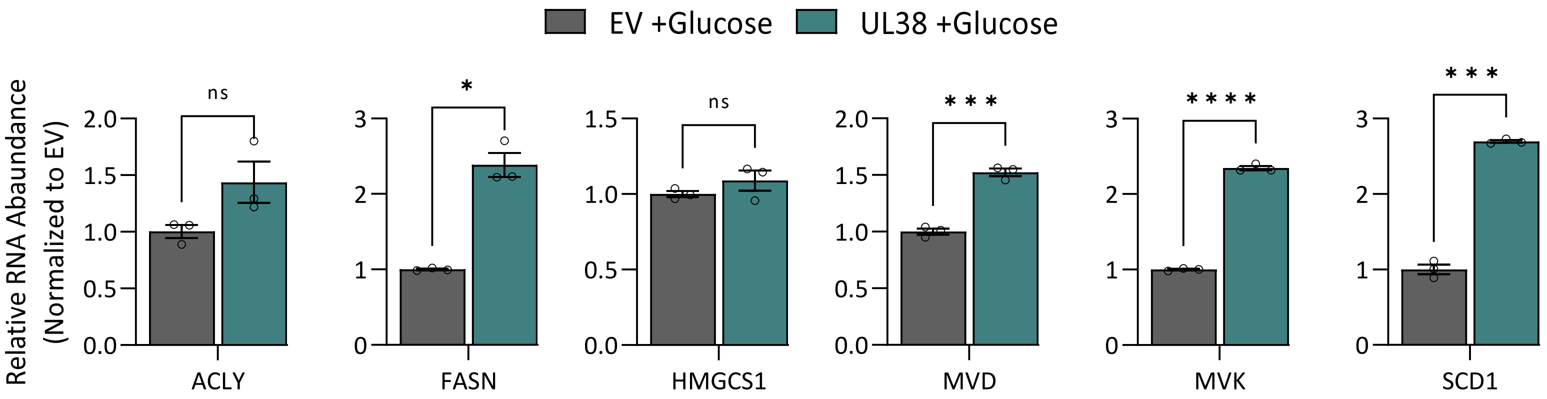

Figure S1. U$_L$38-expression induces the accumulation of SREBF1 target genes. Total RNA was harvested from MRC-5 fibroblasts stably expressing either U$_L$38 or an empty vector (EV) and analyzed by qPCR for RNA abundance. Values are means ± SD (n=3). Brown-Forsythe One-way ANOVA with Welch's correction was used to determine significance. * p<0.05, ** p<0.005, *** p<0.0005, **** p<0.00005

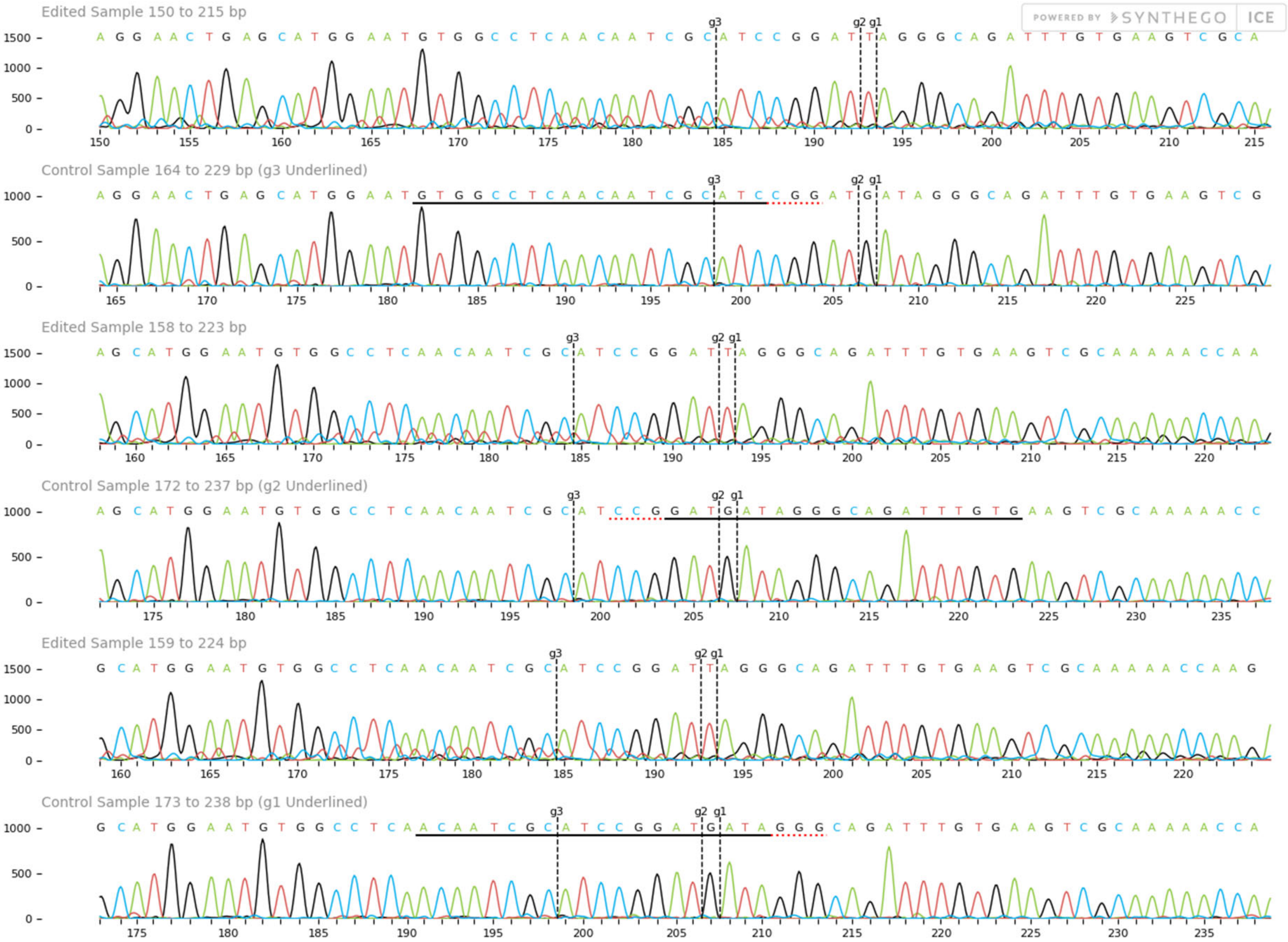

Figure S2. ICE analysis confirms monoclonal TSC2 knockout cells. Genomic DNA was harvested from TSC2 KO cells, PCR amplified, and Sanger sequenced. The DNA traces were then compared to DNA from unedited cells using Inference of CRISPR Edits (ICE) software, which determined the population of cells present to have a 2 base pair deletion at guide 2 cut site.

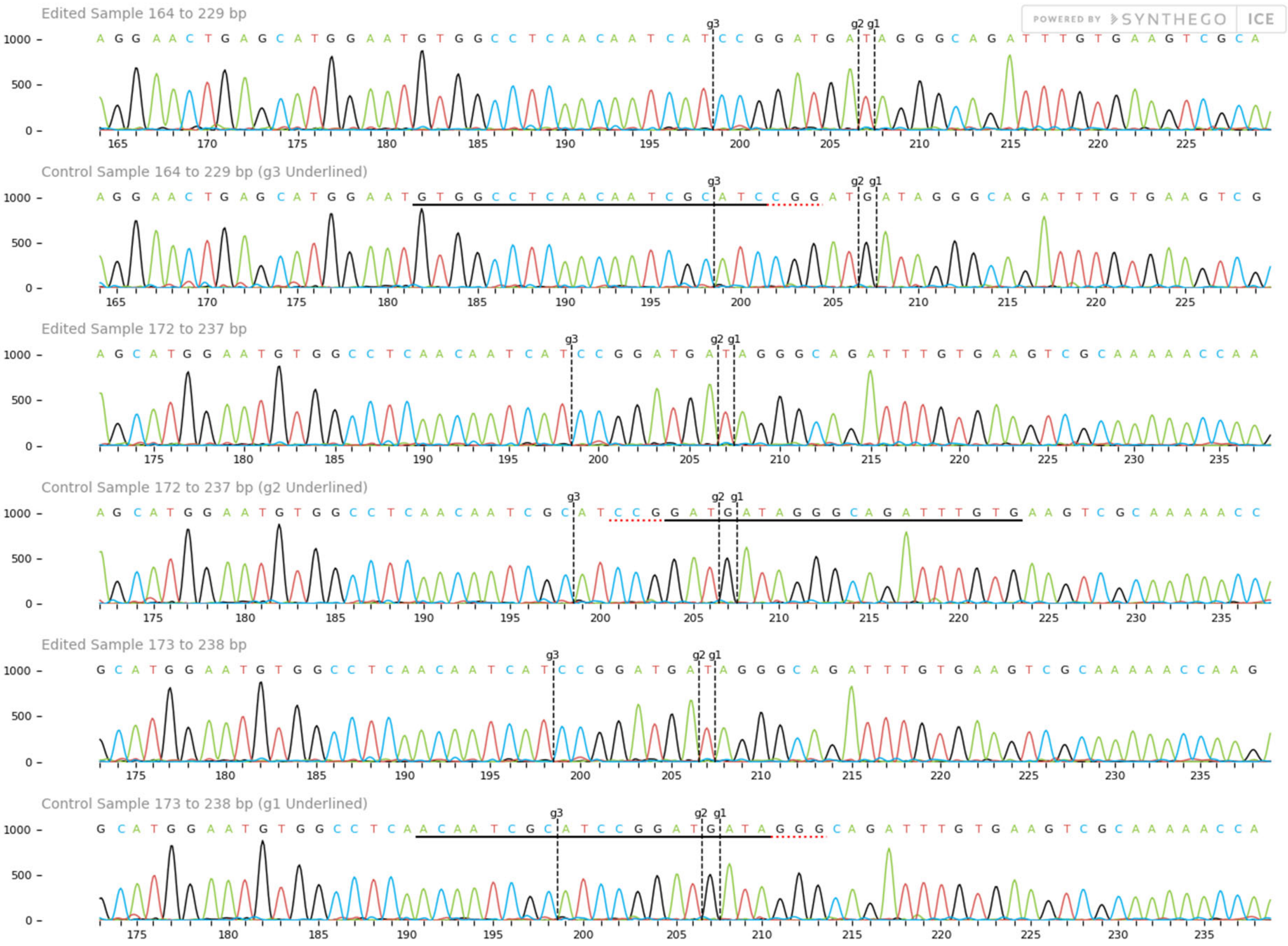

Figure S3. ICE analysis confirms monoclonal TSC2 knockout cells. Genomic DNA was harvested from TSC2 KO cells, PCR amplified, and Sanger sequenced. The DNA traces were then compared to DNA from unedited cells using Inference of CRISPR Edits (ICE) software, which determined the population of cells present to have a 2 base pair deletion at guide 3 cut site.

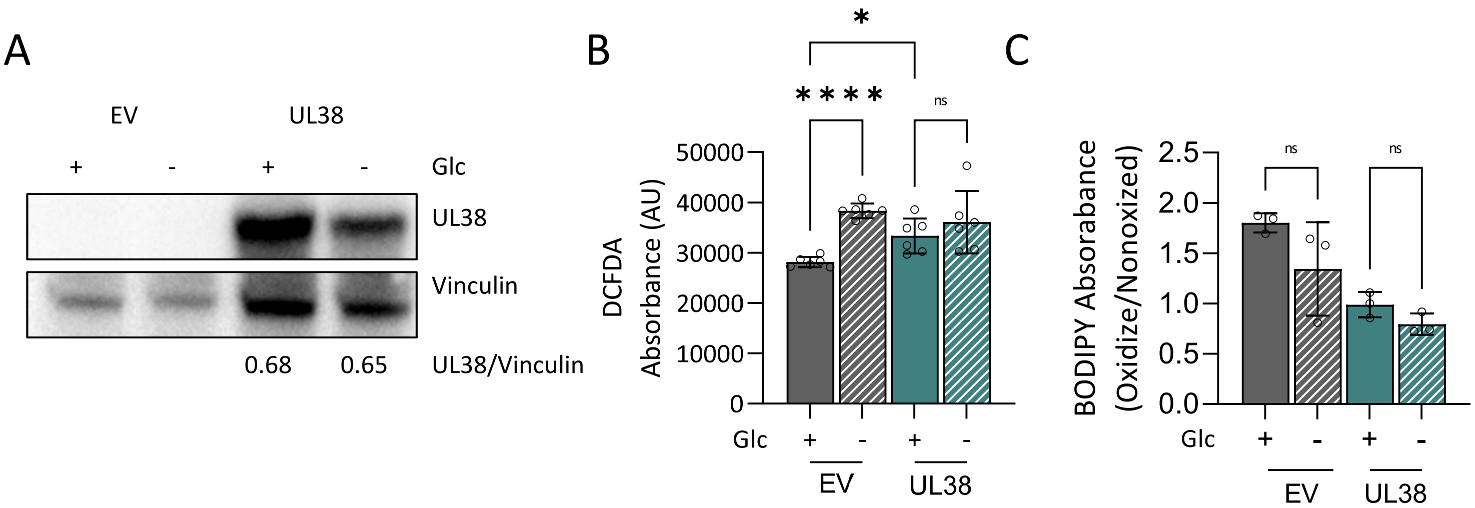

Figure S4. UL38 expression does not increase lipid oxidation or ROS generation during glucose withdrawal. (A-C) Empty vector (EV) and wild-type U_L38 (U_L38) expressing fibroblasts were given glucose-containing or glucose free media for 24 hours. **(A)** At 24 hours, proteins were harvested for western blot analysis **(B)** At 24 hours, DCFDA was used to measure ROS generation. Values are means ± SD (n=6). **(C)** BODIPY 521/600 was used to measure lipid oxidation. Values are means ± SD (n=3). Brown-Forsythe One-way ANOVA with Welch's correction was used to determine significance. * $p<0.05$, ** $p<0.005$, *** $p<0.0005$,

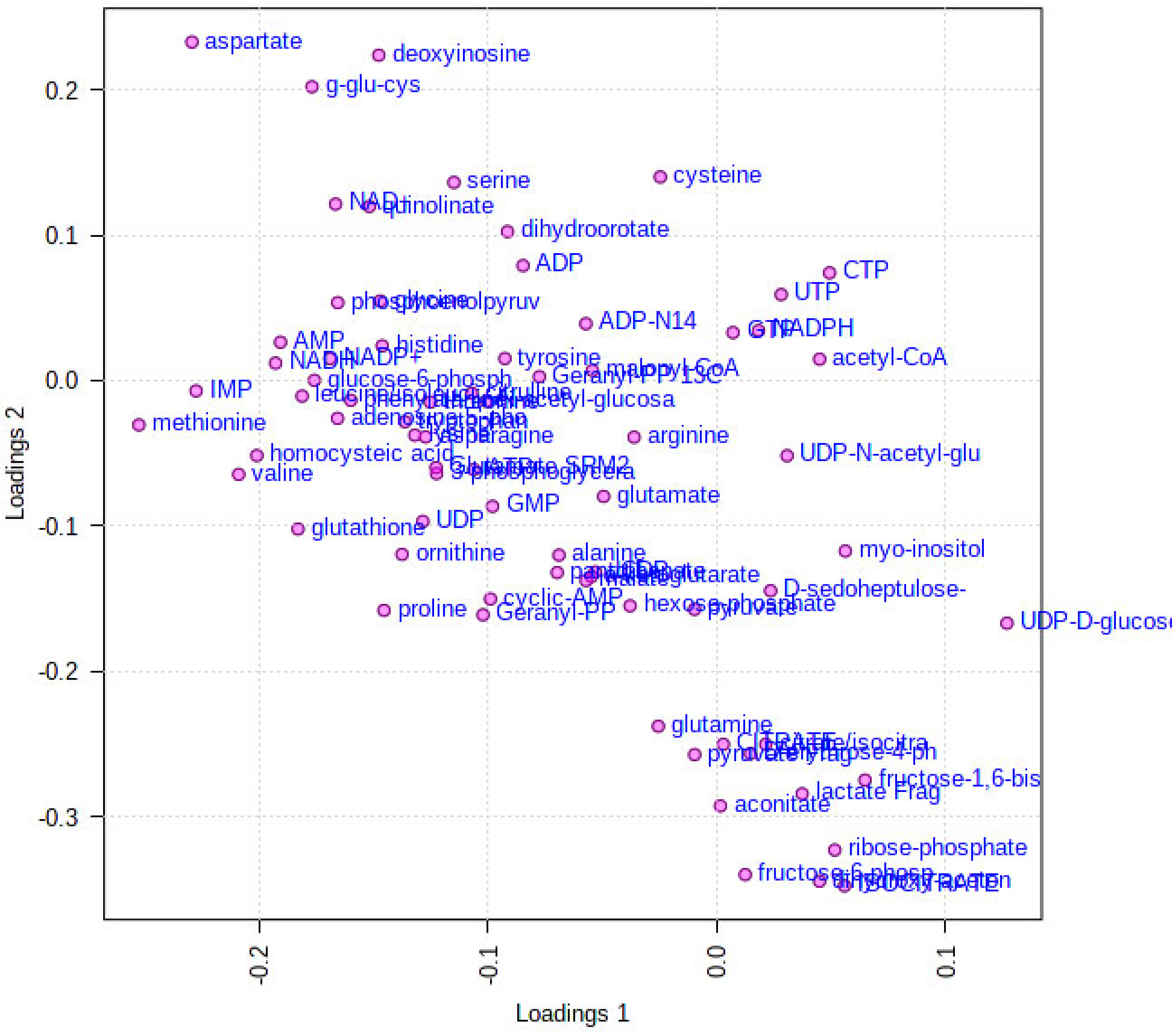

Figure S5. U$_L$38 expression induces significant metabolic alterations to the cellular response to glucose limitation. MRC-5 fibroblasts expressing an empty vector (EV) or wild-type U$_L$38 (U$_L$38) were grown to confluence and cell-cycle synchronized with serum-free medium for 24 hours. Media were replaced with glucose containing or glucose free media for 24 hours and cellular metabolites were harvested and analyzed via LC-MS/MS. Figure depicts the loading plot used in conjunction with Fig. 5 principal component analysis.

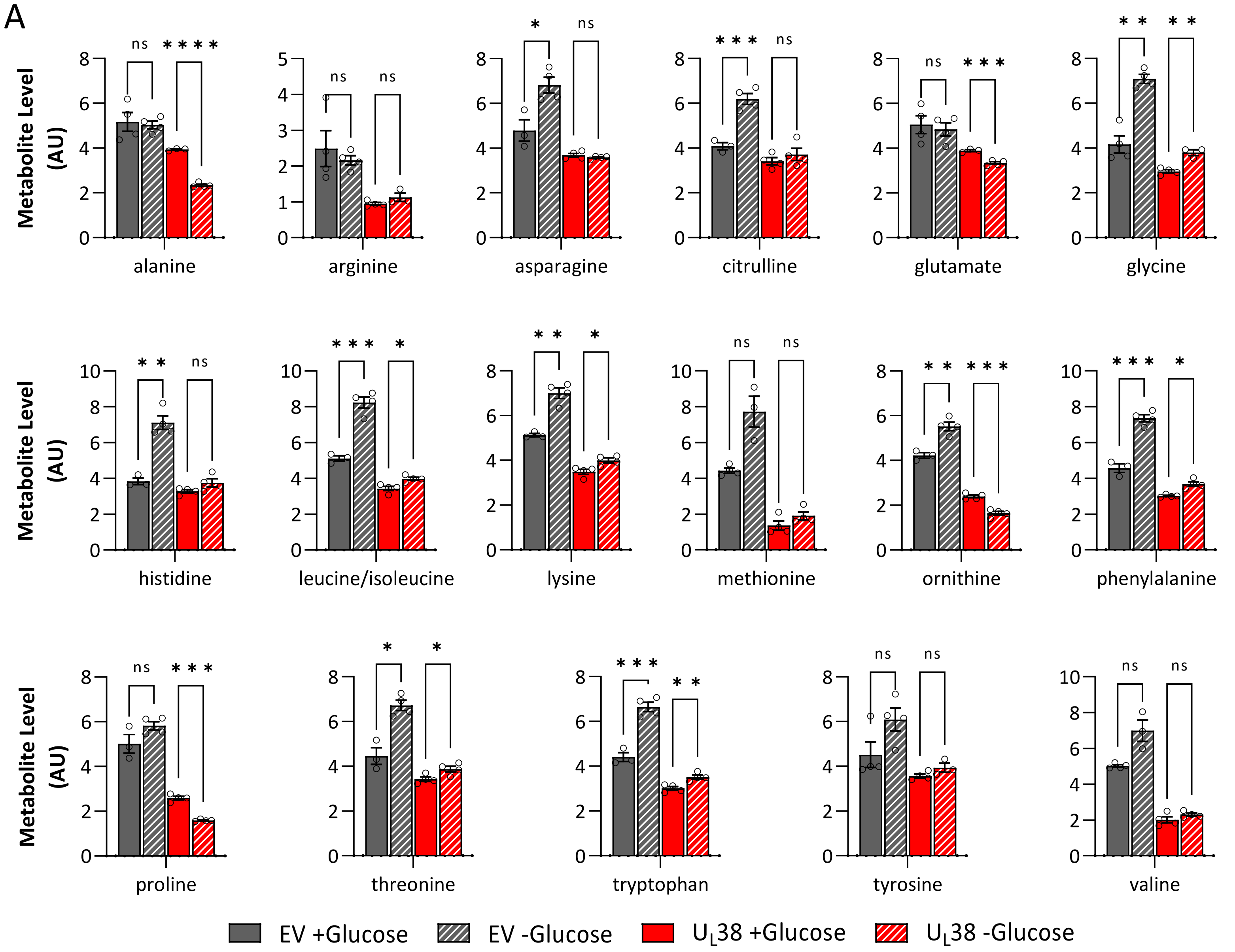

Fig S6. U$_L$38 expression significantly alters intracellular amino acid abundance during glucose limitation. Fibroblasts expressing an empty vector (EV) or wild-type U$_L$38 (U$_L$38) were grown to confluence and cell-cycle synchronized with serum-free medium for 24 hours. Media were replaced with glucose-containing or glucose free media for 24 hours and cellular metabolites were harvested and analyzed via LC-MS/MS. Grey = EV, Red = U$_L$38, Striped = no glucose. Values are mean ± SD (n=4). Brown-Forsythe One-way ANOVA with Welch's correction was used to determine significance. * $p<0.05$, ** $p<0.005$, *** $p<0.0005$, **** $p<0.00005$

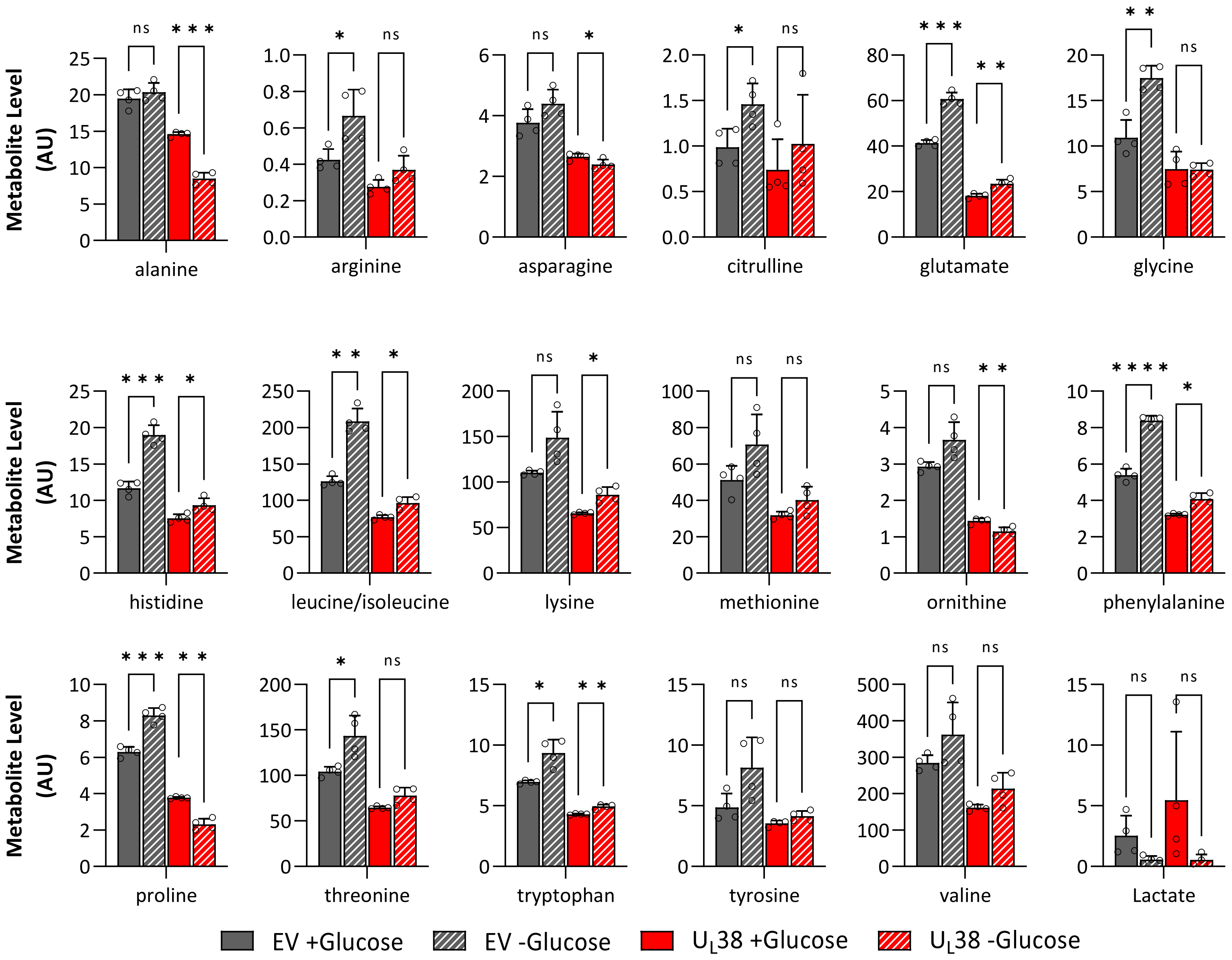

Fig S7. U_L38 expression significantly alters media amino acid concentrations during glucose limitation. Fibroblasts expressing an empty vector (EV) or wild-type U_L38 (U_L38) were grown to confluence and cell-cycle synchronized with serum-free medium for 24 hours. Media were replaced with glucose-containing or glucose free media for 24 hours and media were harvested and analyzed via LC-MS/MS. Grey = EV, Red = U_L38, Striped = no glucose. Values are mean ± SD (n=4). Brown-Forsythe One-way ANOVA with Welch's correction was used to determine significance. * $p<0.05$, ** $p<0.005$, *** $p<0.0005$, **** $p<0.00005$

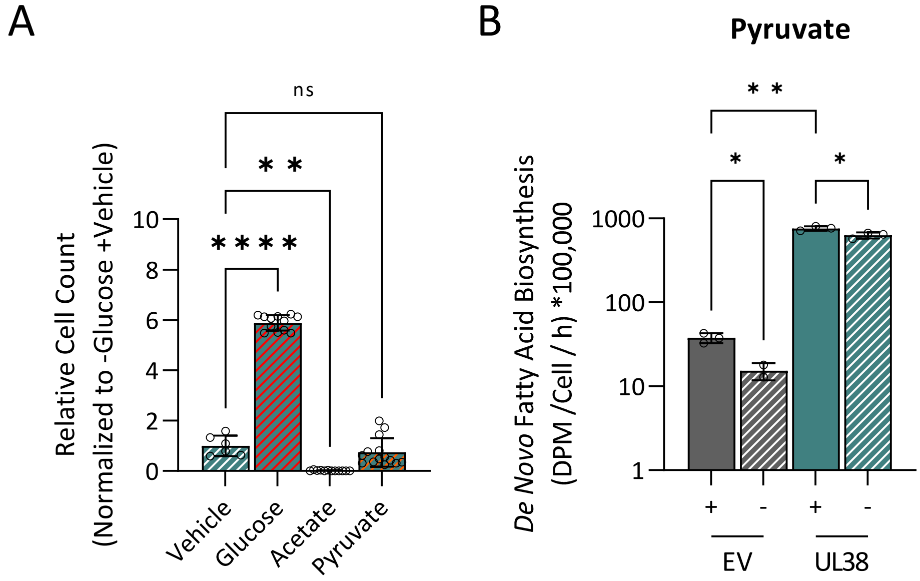

Fig S8. Acetate does not rescue U_L38-mediated glucose sensitivity. (A) U_L38 expressing fibroblasts were grown to confluence and cell-cycle synchronized with serum-free medium for 24 hours. Media were replaced with glucose free media supplemented with PBS, glucose (4.5 mg/mL), acetate (20 μM) or pyruvate (1 mM). At 72 hours post glucose-challenge cellular nuclei were stained, imaged, and counted. Values are mean ± SD (n=6). **(B)** MRC-5 fibroblasts expressing an empty vector (EV) or wild-type U_L38 (U_L38) were grown to confluence and challenged with glucose free medium for 18 hours at which time cells were labeled with ^{14}C-pyruvate for 6h. Lipids were subsequently extracted, saponified and the radioactivity in the lipogenic fraction scintillation counted. Values are means ± SD (n=3). Brown-Forsythe One-way ANOVA with Welch's correction was used to determine significance. * p<0.05, ** p<0.005, *** p<0.0005, **** p<0.00005.
